# Test–Retest Reliability of Balance Parameters Obtained with a Force Platform in Individuals with Chronic Obstructive Pulmonary Disease

**DOI:** 10.3390/jfmk11010024

**Published:** 2026-01-01

**Authors:** Igor Lopes de Brito, Walter Sepúlveda-Loyola, Larissa Araújo de Castro, Leidy Tatiana Ordoñez-Mora, Ademilson Julio da Silva Junior, Vanessa S. Probst

**Affiliations:** 1 Program of Master’s and Doctoral Degree in Rehabilitation Sciences, University of Londrina (UEL), Londrina 86038-440, Brazil; ilbrito@hotmail.com (I.L.d.B.); larissa.decastro@yahoo.com.br (L.A.d.C.); vanessaprobst@uel.br (V.S.P.); 2 Faculty of Health and Social Sciences, Center for Research in Biological and Chemical Sciences, Universidad de Las Américas (UDLA), Santiago 7500975, Chile; wsepulveda@udla.cl; 3 Physiotherapy Program, Faculty of Health, Universidad Santiago de Cali, Santiago de Cali 760035, Colombia; 4 Residency Program in Respiratory Physiotherapy, State University of Londrina (UEL), Londrina 86038-440, Brazil; ademilson.silvajr@gmail.com

**Keywords:** aged, balance, postural control, chronic obstructive pulmonary disease, pulmonary rehabilitation

## Abstract

**Background**: Impaired postural balance is a common feature in individuals with chronic obstructive pulmonary disease (COPD), increasing their risk of falls. This study aimed to evaluate the test–retest reliability of force platform parameters used to assess postural balance in individuals with COPD. **Methods**: A test–retest reliability study was conducted with participants diagnosed with moderate to severe COPD. Each participant completed two standardized balance assessments on a force platform, separated by a seven-day interval. Center of pressure (COP) parameters—including sway area, mean velocity, and path length—were analyzed under eyes-open and eyes-closed conditions. Reliability was determined using intraclass correlation coefficients (ICC), standard error of measurement (SEM), and coefficient of variation (CV). Correlations were performed between force platform parameters, the Timed Up and Go (TUG) test, and the Downton Fall Risk Scale. **Results**: Twenty individuals with COPD (mean age: 67.8 ± 6.1 years; forced expiratory value in the first second: 54 ± 12% predicted) were evaluated. The COP parameters demonstrated good to excellent test–retest reliability (ICC = 0.82–0.95) across all conditions, with low measurement error (SEM < 10%). Moderate correlations were found between force platform parameters and both TUG performance (r = 0.52–0.67) and Downton scores (r = 0.48–0.61). **Conclusions**: Force platform measurements show high reliability for assessing postural balance in individuals with COPD. These findings support the use of objective balance assessment tools in pulmonary rehabilitation and for monitoring fall risk in this population.

## 1. Introduction

Chronic obstructive pulmonary disease (COPD) is primarily a pulmonary disorder characterized by persistent airflow limitation; however, it also involves extrapulmonary manifestations such as altered body composition and skeletal muscle dysfunction [1,2]. Previous studies have demonstrated that individuals with COPD exhibit postural control impairments that lead to balance disturbances [3,4,5]. Smith et al. reported that the increased respiratory workload in COPD leads to trunk oscillation, resulting in poorer postural stability, particularly during standing balance tasks [6].

These impairments in postural control, combined with muscular alterations, make individuals with COPD more susceptible to falls [7]. Prospective studies have reported an annual incidence of approximately 1.2 falls per patient with COPD [8,9], which is a rate significantly higher than that observed in healthy older adults (0.24 falls per year) [10]. Multiple intrinsic factors have been identified as contributors to fall risk in this population, including advanced age, polypharmacy, reduced gait speed, muscle weakness, and balance impairment [11]. Tinetti et al. showed that healthy older adults with one fall risk factor have a 27% likelihood of falling, increasing to 78% when individuals present with four or more risk factors [12].

Given that postural balance relies on the integration of multiple sensory and motor systems, the use of subjective clinical balance assessments may introduce measurement bias. To address this limitation, objective tools such as force platform posturography have been proposed as a more precise method to quantify balance performance [13]. Among the variables in force platform posturography, the center of pressure (COP) defined as the point of application of the ground reaction force vector—is the most used parameter for postural stability assessment [14,15]. However, as COP parameters originate from biological systems, they naturally exhibit variability, which may affect their reliability and validity [13,16]. Although COP parameters are well established in general balance research [17], evidence regarding their reproducibility specifically in individuals with COPD populations remains limited. Prior studies involving COPD populations have employed force platforms to quantify balance performance [18,19,20], yet none, to our knowledge, have systematically examined the test–retest reliability of COP-derived metrics in this group. This gap is particularly relevant given the increasing use of objective balance assessments in clinical trials and rehabilitation settings, especially within fall-prevention contexts.

Therefore, the primary objective of this study was to assess the test–retest reliability of COP parameters obtained from a force platform in individuals with COPD. A secondary aim was to examine the associations between these objective balance measures, functional performance, and fall risk.

## 2. Materials and Methods

### 2.1. Study Design and Ethical Considerations

This was a test–retest reliability study developed at the University Hospital of Paraná, Londrina. The project was approved by the ethic committee of the Londrina State University, UEL, Brazil (PP/0007/14). All subjects were fully informed of the study procedures and provided written informed consent before enrollment. The study was conducted in accordance with the Strengthening the Reporting of Observational Studies in Epidemiology (STROBE) statement: guidelines for reporting observational studies [21].

### 2.2. Participants

The determination of the sample size in this reliability analysis was guided by evidence from prior studies involving comparable balance assessments, which indicated that at least 20 participants were necessary [16]. Accordingly, a total of 20 subjects were included, a number that provided a statistical power greater than 0.99 for identifying reliability coefficients exceeding the predefined criterion. This estimation was supported by the classical model of Walter, Eliasziw, and Donner, commonly applied for evaluating intra-class correlation coefficients (ICC) in test–retest reliability research. The calculation assumed a minimum acceptable reliability coefficient (ρ_0_) of 0.60, two repeated measurements per subject (k = 2), a significance level of α = 0.05, and an expected ICC higher than the specified lower bound [22].

Participants were eligible if they had a confirmed diagnosis of chronic obstructive pulmonary disease (COPD) based on the Global Initiative for Obstructive Lung Disease (GOLD) criteria [1], had not experienced an exacerbation within the preceding three months, and presented no severe comorbid conditions that could compromise test performance. Additionally, individuals who had engaged in structured physical training programs during the previous year were not considered eligible. Participants were excluded if they were unable to complete the assessment procedures or if they chose to withdraw from the study at any stage.

### 2.3. Data Collection Procedures

The experimental procedures were distributed across three separate sessions. On the first day, participants underwent anthropometric assessment, spirometric evaluation of pulmonary function, determination of fall risk using the Downton Fall Risk Index, cognitive screening with the Mini-Mental State Examination, and familiarization with the force platform. The second session, conducted three days later, involved the initial postural control assessment (test) on the force platform, following a randomized order of four balance tasks. The third session, held seven days after the second visit, consisted of a repeated postural control assessment (retest) using the same task sequence, together with the Timed Up and Go test to evaluate functional balance. All evaluations were administered by the same examiner, and participants were instructed to maintain their regular routines throughout the study period. Further methodological details are described below.

#### 2.3.1. Pulmonary Function

Standard spirometry (Spiropalm, COSMED, Italy) was conducted for diagnostic confirmation and disease severity classification, in accordance with international guidelines [23]. Prior to each testing session, the spirometer was calibrated following the manufacturer’s specifications. Assessments were performed with participants seated, using a nose clip and a disposable mouthpiece to ensure an adequate seal and reduce air leakage. A minimum of three forced expiratory maneuvers were obtained from each participant, with standardized verbal encouragement provided to promote maximal effort. Flow–volume curves were monitored in real time to confirm acceptability and reproducibility criteria. The highest recorded values of forced vital capacity (FVC) and forced expiratory volume in the first second (FEV_1_), as well as their ratio (FEV_1_/FVC), were used for analysis. Reference values established for the Brazilian population were applied for disease classification [24].

#### 2.3.2. Risk of Falling

The Downton fall risk index was used to assess the risk of falling. This instrument uses five criteria to assess risk of falling: (1) previous falls, (2) medication usage, (3) sensory deficits, (4) mental state and (5) gait quality. The score varies from zero to eleven with a cutoff point greater than or equal three that indicates high risk of falling [25,26].

#### 2.3.3. Cognitive Status

The Mini Mental State Examination (MMSE) was administered to evaluate participants’ global cognitive status. This standardized exam consists of 30 items distributed across five cognitive domains: (1) orientation; (2) registration of verbal information; (3) attention and calculation; (4) word recall; and (5) language and praxis [27].

#### 2.3.4. Postural Control

Postural control was evaluated using a force platform (BIOMEC 400, EMG System do Brasil Ltda., São José dos Campos, SP, Brazil), a triaxial measurement system equipped with four strain gauges capable of detecting the three components of the ground reaction force (Fx, Fy, and Fz), as well as the moments around the anteroposterior and mediolateral axes. This configuration enabled precise computation of the center of pressure (COP) displacements in both the anteroposterior and mediolateral planes.

Force signals were sampled at 100 Hz, in accordance with international recommendations for the assessment of static postural balance, ensuring adequate temporal resolution for the analysis of postural sway. Postural balance was assessed under four experimental conditions: standing with feet hip-width apart and eyes open (FHEO), standing with feet hip-width apart and eyes closed (FHEC), standing with feet together such that the medial malleoli were in contact (short stance base, SSB), and one-legged stance (OLS). Participants completed three 60-s trials for the FHEO, FHEC, and SSB conditions, with 30-s rest intervals between trials. For the OLS condition, three 30-s trials were performed on the preferred leg, separated by 30-s rest intervals. The mean value of the three trials was used for each condition in subsequent analyses.

During testing, participants stood barefoot, with arms relaxed alongside the body and gaze fixed on a visual target placed two meters ahead at eye level. Data acquisition and processing were conducted in a biomechanics laboratory (LAFUP–UNOPAR) under controlled environmental conditions (adequate lighting and minimal background noise). All data were collected and analyzed by licensed physiotherapists with advanced training and extensive experience in postural control and biomechanical assessment, ensuring methodological rigor and data reliability. From the COP trajectories, balance parameters were derived, including the 95% confidence ellipse area of the COP (cm^2^), as well as mean velocity (cm/s) and mean frequency (Hz) in the anteroposterior and mediolateral directions.

#### 2.3.5. Functional Balance

The Timed Up and Go (TUG) test was used to evaluate functional balance [28]. Participants were instructed to rise from a standardized chair, walk three meters at their self-selected pace, turn around, return to the chair, and sit down. Two attempts were completed, with a one-minute rest period between trials. The fastest performance was selected as the outcome for analysis [29].

### 2.4. Statistical Analysis

Statistical analyses were conducted using Microsoft Excel, SPSS Statistics version 20.0 (SPSS Inc., Chicago, IL, USA), and GraphPad Prism version 6.0 (GraphPad Software Inc., San Diego, CA, USA). Data distribution was analyzed with Shapiro–Wilk test. This value measures the amount of error that can be attributed to sample and can also be considered as an estimate of the variability around the population mean. The percentage (%) of SEM was also calculated relative to mean value from test and retest. A complementary analysis of data using the Bland–Altman plot was also performed. In addition, a comparison between test and retest was conducted using the paired *t* test or Wilcoxon when necessary. Test–retest reliability was assessed using Intraclass Correlation Coefficients (ICC), adopting a two-way mixed-effects model with absolute agreement. ICC values were interpreted according to established thresholds: <0.50 poor, 0.50–0.75 moderate, 0.75–0.90 good, and >0.90 excellent reliability. Standard Error of Measurement (SEM) and Minimal Detectable Change (MDC90) were also calculated to quantify measurement precision. Finally, Pearson or Spearman correlation coefficients were computed to determine the relationship between the variables (COP, TUG, fall risk and number of falls). Statistical significance was determined as *p* < 0.05. A post hoc power analysis for ICC was performed following the method described by Walter, Eliasziw, and Donner (1998) [22], confirming that the sample size of 20 participants provided very high statistical power (>0.99) to detect reliability coefficients exceeding the minimum acceptable threshold (ρ_0_ = 0.60).

## 3. Results

### 3.1. Participant Characteristics

The study sample consisted of 20 individuals with moderate to severe COPD, with a mean age of 71 ± 6 years and a male predominance (65%). The mean FEV_1_ was 50.1 ± 16% of predicted, and the average FEV_1_/FVC ratio was 0.58 ± 0.11. According to the GOLD classification, most participants were categorized as stage II (*n* = 11), followed by stage III (*n* = 7) and IV (*n* = 2). The characteristics of the individuals with COPD are presented in Table 1.

### 3.2. Test–Retest Reliability of Force Platform Parameters

Table 2 presents the intraclass correlation coefficients (ICC), 95% confidence intervals (CI), standard error of measurement (SEM), and percentage of SEM for the COP parameters across the four balance tasks. All tested conditions demonstrated good to excellent test–retest reliability (ICC range: 0.85 to 0.97). The highest reliability was observed for anteroposterior (Vel-AP) and mediolateral velocity (Vel-ML), particularly in the short base (SSB) and one-legged stance (OLS) conditions. For example, Vel-ML during OLS showed an ICC of 0.97 with a SEM of only 0.42. The COP area showed slightly lower ICC values, particularly in the OLS condition (ICC = 0.85), though still within the acceptable range. SEM values remained low across all tasks, with percentage SEMs below 35%, indicating minimal measurement error and high measurement precision.

### 3.3. Comparison Between Test and Retest Values

To evaluate consistency between testing days, force platform parameters from the initial and follow-up sessions were compared (Table 3). No statistically significant differences were found between test and retest measurements for any of the COP parameters (all *p* > 0.05), indicating stable performance across days.

**Figure 1:**
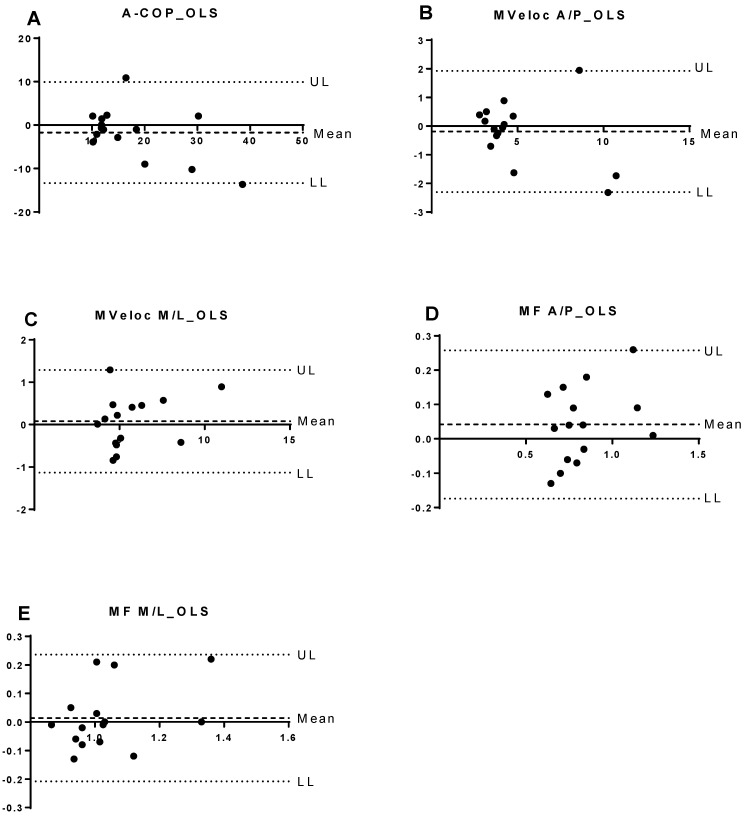
Bland–Altman plots of the difference between test and retest of force platform parameters with the mean value found in one-legged stance. Legend. The solid horizontal line represents the mean difference between measurements, while the dashed lines indicate the upper (UL) and lower (LL) limits of agreement (±1.96 SD). Panels correspond to: (**A**) COP area (A-COP_OLS), (**B**) mean anteroposterior velocity (MVeloc A/P_OLS), (**C**) mean mediolateral velocity (MVeloc M/L_OLS), (**D**) mean anteroposterior frequency (MF A/P_OLS), and (**E**) mean mediolateral frequency (MF M/L_OLS). The plots show good agreement and minimal systematic bias, indicating stable measurement performance across test sessions. Abbreviation: OLS: one-legged stance; UL: upper limit; LL: lower limit; COP-area: 95% confidence ellipse area of center of pressure; Vel-AP: anteroposterior mean velocity; Vel-ML: mediolateral mean velocity; Freq-AP: anteroposterior mean frequency; Freq-ML: mediolateral mean frequency.

The median COP area during eyes-open stance (FHEO) was 2.7 cm^2^ [1.3–5.1] on the first test and 2.14 cm^2^ [1.5–4.8] on the retest (*p* = 0.49). Similar stability was observed for velocity and frequency parameters across all conditions. These results are visually supported by Figure 1, which displays Bland–Altman plots of test–retest differences during the one-legged stance task. The plots reveal that most values lie within the limits of agreement, with greater dispersion observed in the anteroposterior direction.

### 3.4. Correlation Between Balance Parameters and Functional Outcomes

Correlations between COP variables and clinical measures (TUG, number of falls, and Downton Fall Risk Index) are detailed in Table 4. Significant moderate-to-strong correlations were found between functional balance (TUG) and velocity parameters in all balance conditions, especially in Vel-AP (r = 0.64 to 0.80, *p* < 0.05). Moreover, significant correlations were also observed between Vel-AP and Vel-ML and the Downton Fall Risk Index, particularly in the FHEO, FHEC, and SSB conditions (r = 0.51 to 0.68, *p* < 0.05).

## 4. Discussion

The present study is the first to evaluate the reliability of COP parameters derived from a force platform across four balance tasks in individuals with COPD. The findings demonstrated excellent reliability for all COP parameters. Consistent with the results reported by Smith et al. [6], our results indicate that balance was better preserved in the anteroposterior direction among participants with COPD. Furthermore, this study is the first to identify a significant correlation between COP parameters obtained from the force platform and performance on a functional balance test.

The study of da Silva et al. [16] investigated the reliability of the force platform to assess balance in twenty healthy elderly. The condition of balance assessment was the one-legged stance with eyes opened. The authors performed the test and retest in two sessions separated by a maximum of two weeks. The ICC values observed by da Silva et al. [16] varied between 0.40 and 0.85, which somehow contrasts with the ICC observed in the present study, all higher than 0.85. The present study assessed balance in different conditions to verify the COP oscillation behavior, which enabled a broader assessment of reliability, reproducing more positions adopted during activities of daily living.

Gasq et al. [17] examined the reliability of force platform measurements for assessing balance in individuals with post-stroke hemiparesis. Balance was evaluated under two visual conditions—eyes open and eyes closed—while participants stood in a bipedal stance with heels apart. Twenty participants were assessed on two separate occasions, one week apart. The authors reported intraclass correlation coefficient (ICC) values ranging from 0.71 to 0.97, which are comparable to those observed in the present study (0.85–0.98).

The high ICC values observed in both studies may be explained by the inclusion of participants with chronic diseases, as chronic conditions are recognized intrinsic risk factors for falls [11]. Moreover, the participants demonstrated postural instability, reflected by COP sway displacement with low variability. This phenomenon can be interpreted through the inverted pendulum theory of postural control [10], which defines the limits of stability as the maximal range of oscillation that allows the body to remain upright without the need for corrective movements. Accordingly, greater COP oscillation in individuals with chronic diseases, compared with healthy older adults, brings body sway closer to these stability limits, thereby reducing the available area for COP variability. Notably, the prevalence of falls among individuals with COPD (40%) is similar to that observed in populations with neurological disorders (42%), suggesting that comparable postural control mechanisms may underlie balance impairments across different chronic conditions [18,19,30].

In the present study, individuals with COPD exhibited poorer balance, as indicated by higher center of pressure (COP) values, particularly in the mediolateral direction. This finding is consistent with previous studies [6,17]. Moreover, the high ICC and low SEM values observed in this direction (Table 2) warrant particular attention. The coexistence of poorer balance with high reliability may be explained by the inverted pendulum theory, which suggests that in the mediolateral plane, individuals with COPD tend to sway near their limits of stability and, consequently, display a smaller area available for postural oscillation.

The present study is the first to demonstrate correlations between the force platform parameters and functional balance test. Gil et al. [31] also investigated associations between force platform parameters and two functional balance assessments: the traditional one-leg stance test and an agility/dynamic balance test. The former measures the time an individual can maintain a one-legged stance, while the latter quantifies agility and dynamic balance by recording the total time required to sit, stand, and walk as quickly as possible around two cones. Gil et al. [31] observed weak correlations between the force platform parameters and functional balance tests. In the present study, however, moderate to strong correlations were observed between the force platform and TUG, a widely used functional balance assessment. Although the functional balance test employed here differed from those used by Gil et al. [31], both rely on the time required to complete the task as the primary outcome, present a more functional profile, and capture aspects of dynamic balance. Additionally, regarding static balance, the present study also evaluated multiple postural conditions, whereas Gil et al. [31] assessed balance exclusively in a single condition (one-legged stance). This broader range of conditions, feet hip-width apart with eyes opened and closed, a reduced base of support, and one-legged stance, allowed us to observe strong correlations between static and functional balance across most conditions. However, in the one-legged stance condition, no significant association between the force platform parameters and TUG performance was found, consistent with the findings of Gil et al. [31].

Despite all efforts, this study presents some limitations. A potential concern is that the sample size was too small. However, based on the high reliability coefficients observed, the sample size was sufficient to address the study objectives and supports the robustness of the findings. Another point of consideration might be that most of the sample was composed of men; however, regarding sex distribution, the predominance of male participants reflects the epidemiological profile of COPD both in Brazil [32] and globally [1]. Additionally, the prevalence of falls in the studied sample (0.34 falls per subject per year) was lower than that typically reported in the COPD literature (approximately 1.2 falls per subject per year). This discrepancy limits the generalizability of our findings to the broader COPD population. It is plausible that this lower fall prevalence is related to the comparatively preserved exercise capacity and higher levels of daily physical activity reported in Brazilian subjects with COPD [33]. Finally, it could be argued that changes in medication might have influenced the results. However, no participant experienced any modification in pharmacological therapy during the assessment period. All individuals remained clinically stable and maintained their usual COPD treatment regimen, ensuring that medication effects did not introduce variability into the measurements.

Given the growing recognition of balance impairments in individuals with COPD, the findings of the present study provide relevant contributions to both clinical practice and research. Our results indicate that force platforms represent a reliable tool for the assessment of static balance in this population. Accordingly, their use may support the accurate identification of individuals with balance deficits, facilitating appropriate referral to targeted rehabilitation interventions. Accordingly, their use may support the accurate identification of individuals with balance deficits, facilitating appropriate referral to targeted rehabilitation interventions.

## 5. Conclusions

This study demonstrated that the force platform is a reliable instrument for assessing static postural balance in individuals with COPD. The high test–retest reliability observed across center of pressure parameters indicates that this tool provides consistent and reproducible measurements under standardized conditions. Furthermore, the significant correlations between force platform outcomes, functional balance performance, and fall risk scores highlight its potential clinical applicability in the comprehensive evaluation of individuals with COPD. Incorporating objective balance assessment tools, such as the force platform, into pulmonary rehabilitation programs may enhance the identification of balance impairments, guide targeted interventions, and contribute to fall prevention strategies. Future research should explore the responsiveness of these measures to rehabilitation interventions and their predictive value for real-world fall events in this population.

## Figures and Tables

**Table 1:** Characteristics of the sample.

**Variables**	
*n* (M/F)	20 (13/7)
Age (years)	71 ± 6
FEV_1_ (% predicted)	50.1 ± 16
FEV_1_/FVC	0.58 ± 0.11
GOLD classification (I/II/III/IV)	0/11/7/2
BMI (kg/m^2^)	26 ± 5
TUG (s)	12.6 ± 3.3
Number of falls in the last year	0.35 ± 0.9
Mini Mental State Examination (score)	25.79 ± 3.61

Explanations: M: Male; F: Female; FEV_1_: Forced expiratory volume in the first second; % predicted: % of the predicted value according to Pereira et al. 2007 [24]; FVC: Forced vital capacity; GOLD: Global Initiative for Chronic Obstructive Lung Disease; GOLD I = FEV_1_/FVC < 0.70 with FEV_1_ ≥ 80% of predicted; GOLD II = FEV_1_/FVC < 0.70 with FEV_1_ ≥ 50% and ≤80% of predicted; GOLD III = FEV_1_/FVC < 0.70 with FEV_1_ ≥ 30% and ≤50%; GOLD IV = FEV_1_/FVC < 0.70 with FEV_1_ < 30%; BMI: Body mass index; TUG: Timed Up and Go test; s: second.

**Table 2:** Intra-class Correlation Coefficient (ICC) of center of pressure data from force platform test–retest.

Tasks	Parameters	ICC	CI 95%	SEM	%SEM
FHEO	COP-area	0.90	0.72–0.97	1.06	33
	Vel-AP	0.96	0.90–0.99	0.15	13
	Vel-ML	0.94	0.80–0.98	0.07	9
	Freq-AP	0.95	0.87–0.98	0.04	11
	Freq-ML	0.88	0.68–0.96	0.05	11
FHEC	COP-area	0.95	0.86–0.98	0.79	24
	Vel-AP	0.95	0.87–0.98	0.15	11
	Vel-ML	0.92	0.80–0.97	0.15	18
	Freq-AP	0.96	0.87–0.98	0.06	17
	Freq-ML	0.90	0.73–0.96	0.04	7
SSB	COP-area	0.94	0.84–0.98	1.56	22
	Vel-AP	0.97	0.92–0.99	0.18	13
	Vel-ML	0.97	0.92–0.99	0.20	12
	Freq-AP	0.91	0.75–0.97	0.08	21
	Freq-ML	0.92	0.77–0.97	0.05	13
OLS	COP-area	0.85	0.55–0.95	2.73	16
	Vel-AP	0.96	0.87–0.99	0.79	16
	Vel-ML	0.97	0.92–0.99	0.42	7
	Freq-AP	0.89	0.66–0.96	0.08	10
	Freq-ML	0.85	0.55–0.95	0.07	7

Explanations: FHEO: standing with feet hip-width apart with eyes opened; FHEC: standing with feet hip-width apart with eyes closed; SSB: standing with a short base; OLS one-legged stance and eyes opened; COP-area: 95% confidence ellipse area of center of pressure; Vel-AP: anteroposterior mean velocity; Vel-ML: mediolateral mean velocity; Freq-AP: anteroposterior mean frequency. Freq-ML: mediolateral mean frequency; ICC: intra-class correlation coefficient; SEM: standard error of measurement.

**Table 3:** Comparison of force platform parameters between test and retest.

Tasks	Parameters	Test	Retest	*p*
FHEO	COP-area	2.7 [1.3–5.1]	2.14 [1.5–4.8]	0.49
	Vel-AP	1.1 [0.9–1.3]	1.0 [0.9–1.4]	0.94
	Vel-ML	0.75 ± 0.18	0.72 ± 0.22	0.38
	Freq-AP	0.34 [0.2–0.4]	0.3 [0.27–0.43]	0.93
	Freq-ML	0.43 [0.4–0.5]	0.45 [0.3–0.62]	0.46
FHEC	COP-area	2.9 [1.3–4.5]	2.25 [1.5–4.7]	0.65
	Vel-AP	1.39 ± 0.45	1.42 ± 0.55	0.60
	Vel-ML	0.79 [0.6–1.0]	0.71 [0.6–1.1]	0.93
	Freq-AP	0.33 [0.3–0.4]	0.36 [0.27–0.46]	0.80
	Freq-ML	0.53 ± 0.16	0.56 ± 0.21	0.28
SSB	COP-area	6.2 [4.3–7.6]	6.83 [4.3–84]	0.47
	Vel-AP	1.24 [1.2–1.5]	1.28 [1.1–1.9]	0.99
	Vel-ML	1.53 [1.4–1.9]	1.57 [1.4–2.0]	0.77
	Freq-AP	0.33 [0.3–0.5]	0.33 [0.24–0.5]	0.84
	Freq-ML	0.34 [0.3–0.4]	0.38 [0.3–0.45]	0.52
OLS	COP-area	13.5 [11.4–21.9]	12.14 [11.7–24.5]	0.32
	Vel-AP	3.9 [3.4–4.9]	3.98 [3.7–5.6]	0.77
	Vel-ML	4.91 [4.4–6.5]	5.06 [4.4–6.1]	0.68
	Freq-AP	0.79 [0.7–0.9]	0.76 [0.71–0.85]	0.20
	Freq-ML	1.02 [0.9–1.1]	1.0 [0.96–1.05]	0.98

Explanations: Data are expressed as mean ± SD and median [IQR]. FHEO: standing with feet hip-width apart with eyes opened; FHEC: standing with feet hip-width apart with eyes closed; SSB: standing with a short base; OLS: one-legged stance; COP-area: 95% confidence ellipse area of center of pressure; Vel-AP: anteroposterior mean velocity; Vel-ML: mediolateral mean velocity; Freq-AP: anteroposterior mean frequency; Freq-ML: mediolateral mean frequency.

**Table 4:** Correlation between the force platform parameters and TUG, number of falls and Downton Fall Risk Index.

Tasks	Parameters	TUG	Falls	Downton Scale
FHEO	COP-area	−0.009	0.13	0.45
	Vel-AP	0.64 *	0.02	0.68 *
	Vel-ML	0.32	0.10	0.62 *
	Freq-AP	0.65 *	−0.14	0.34
	Freq-ML	0.31	0.17	0.08
FHEC	COP-area	0.20	0.12	0.50
	Vel-AP	0.80 *	−0.005	0.63 *
	Vel-ML	0.34	0.04	0.53 *
	Freq- AP	0.64 *	−0.15	0.35
	Freq-ML	0.31	0.15	0.05
SSB	COP-area	0.30	0.24	0.51 *
	Vel-AP	0.72 *	−0.006	0.51 *
	Vel-ML	0.64 *	−0.20	0.42
	Freq-AP	0.64 *	−0.21	0.30
	Freq-ML	0.60 *	−0.10	0.34
OLS	COP-area	0.10	−0.20	0.0002
	Vel-AP	0.45	−0.10	0.36
	Vel-ML	−0.04	−0.2	0.32
	Freq-AP	0.31	−0.08	0.36
	Freq-ML	0.18	−0.07	0.40

Explanations: FHEO: standing with feet hip-width apart with eyes opened; FHEC: standing with feet hip-width apart with eyes closed; SSB: standing with a short base; OLS: one-legged stance; COP-area: 95% confidence ellipse area of center of pressure; Vel-AP: anteroposterior mean velocity; Vel-ML: mediolateral mean velocity; Freq-AP: anteroposterior mean frequency; Freq-ML: mediolateral mean frequency. * *p* < 0.05.

## Data Availability

The data presented in this study are available on request from the corresponding author. The data are not publicly available due to privacy and ethical restrictions.
